# Acceptability and outcomes of the Percutaneous Endoscopic Gastrostomy (PEG) tube placement- patients' and care givers' perspectives

**DOI:** 10.1186/1471-230X-6-37

**Published:** 2006-11-24

**Authors:** Muhammad K Anis, Shahab Abid, Wasim Jafri, Zaigham Abbas, Hasnain A Shah, Saeed Hamid, Rozina Wasaya

**Affiliations:** 1 Department of Medicine, Section Gastroenterology, Aga Khan University Hospital, Stadium Road, Karachi-74800, Pakistan; 2 Endoscopy Unit Aga Khan University Hospital, Stadium Road, Karachi-74800, Pakistan

## Abstract

**Background:**

Percutaneous endoscopic gastrostomy tube has now become a preferred option for the long-term nutritional support device for patients with dysphagia. There is a considerable debate about the health issues related to the quality of life of these patients. Our aim of the study was to assess the outcome and perspectives of patients/care givers, about the acceptability of percutaneous endoscopic gastrostomy tube placement.

**Methods:**

This descriptive analytic study conducted in patients, who have undergone percutaneous endoscopic gastrostomy tube placement during January 1998 till December 2004. Medical records of these patients were evaluated for their demographic characteristics, underlying diagnosis, indications and complications. Telephonic interviews were conducted till March 2005, on a pre-tested questionnaire to address psychological, social and physical performance status, of the health related quality of life issues.

**Results:**

A total of 191 patients' medical records were reviewed, 120 (63%) were males, and mean age was 63 years. Early complication was infection at PEG tube site in 6 (3%) patients. In follow up over 365 ± 149 days, late complications (occurring 72 hours later) were infection at PEG tube site in 29 (15 %) patient and dislodgment/blockage of the tube in 26 (13.6%). Interviews were possible with 126 patients/caretakers. Karnofsky Performance Score of 0, 1, 2, 3 and 4 was found in 13(10%), 18(14%), 21(17%), 29(23%) and 45(36%) with p-value < 0.001. Regarding the social and psychological aspects; 76(60%) would like to have the PEG tube again if required, 105(83 %) felt ease in feeding, and 76(60%) felt that PEG-tube helped in prolonging the survival. Regarding negative opinions; 49(39 %) felt that the feeding was too frequent, 45(36 %) felt apprehensive about dependency for feeding and 62(49%) were concerned about an increase in the cost of care.

**Conclusion:**

PEG-tube placement was found to be relatively free from serious immediate and long- term complications. Majority of caregivers and patient felt that PEG-tube helped in feeding and prolonging the survival. Studies are needed to assess the real benefit in terms of actual nutritional gain and quality of life in such patients.

## Background

Percutaneous endoscopic gastrostomy (PEG-tube) was first introduced in 1980 as an alternative to nasogastric tubes and surgically placed gastrostomy tubes [1]. It has now become an excellent alternative for the long- term management of patients with dysphagic stroke or for those who are unable to feed themselves with intact gastrointestinal tract [2]. PEG-tube has been found to be a safe and effective procedure and replacing open gastrostomy for long-term enteral nutrition [3]. Endoscopist can place the PEG-tube by choosing any of the three commonly practiced methods namely, the pull technique, push technique or button gastrostomy. Feeding gastrostomy tube can also be placed by interventional radiologists under fluoroscopy or by surgeons through surgery on the anterior abdominal wall [4]. PEG-tube offers greater patient comfort, less frequent complications like displacement and greater improvement in the nutritional status. PEG-tube can remain functional for more than one year or longer and requires replacement through the same opening quite infrequently. The main reason for this advantage is the fact that this tube is made up of Silicon material, an inert substance that has neither local reaction nor any systemic complication [5].

Ever since the inception of the PEG-tube, there is a considerable debate about the health issues related to the quality of life (HRQoL) of the patients who have undergone feeding via PEG tube with underlying chronic illnesses [6,7]. Quantitative assessment of the HRQoL is a difficult task. Most researchers measure HRQoL by asking specific questions pertaining to its most important components that include physical, psychological and social domains of health. Although, the objective dimensions are very much important in defining a patient's degree of health; nonetheless, it is the patient's subjective perceptions and expectations that translate the assessment into the actual quality of life experienced [8,9].

The evaluation of the HRQoL and outcomes of the long term nutritional support via the PEG tube from patients' and care givers' perspective has received little attention, especially in the developing countries [10-12]. In this study, we assessed the clinical outcome, patients and care givers perspective of HRQol in terms of performance status, social and psychological aspect, of those patients who underwent PEG-tube placement in our centre.

## Methods

This is a descriptive analytic study done from January 1998 till December 2004 and follow up was recorded by telephonic interview until March 2005. This study was funded by the Department of Medicine, Aga Khan University Karachi. This study consisted of two components, an analysis of patients' clinical outcomes by reviewing their medical records, and a telephonic interview of patients or their caregivers for the assessment of acceptability and health related quality of life issues, after the verbal consent obtained over on the telephone. They were explained about the publishing of the research for the benefit of the similar patients without the disclosure of any personnel identification.

### PEG-tube placement technique

We used Silicon made PEG 24 Tube (Wilson Cook, Durham, NC, USA) that was made up of an inert substance. This procedure was performed under conscious sedation and antibiotic prophylaxis. The upper gastrointestinal endoscopy involved endoscopic visualization of upper gastrointestinal tract up to the second part of the duodenum and to exclude any intraluminal obstruction. The site for placement of PEG- tube was located via trans-illumination on the abdominal wall, followed by an incision, and placement of a cannula (provided in the PEG-tube kit). A guide wire was threaded in the stomach cavity through that cannula and grasped by a snare forceps. Then guide wire was pulled out from the mouth, through which PEG-tube was tied and then PEG-tube was passed into the stomach cavity by pulling of the guide wire through an incisional hole created in the anterior abdominal wall. PEG- tube was placed on the left upper quadrant of the anterior abdominal wall and secured. Positioning of PEG-tube was confirmed with re-endoscopy of the stomach. Teaching to care giver was given regarding the care of the PEG tube and feeding; using commercially based nutritional feeds, along with home based blenderized diets.

### Assessment of clinical status of PEG-tube placement

A questionnaire was designed to review the demographic data including; age, gender, underlying diagnosis for the PEG tube placement, associated diagnosis, early complications (defined as occurring within 72 hours) and late complications (occurring after 72 hours) including; infection at the local site, bleeding, perforation, and dislodgement of the PEG tube. Follow up was also assessed by reviewing medical records of the visits made to the clinic, emergency room or the endoscopy suit. We looked at the parameters such as the fate of the PEG tube, the reasons for removal or replacement, and the number of times it was placed.

### Assessment of acceptability and health related quality of life (HRQoL) of the PEG-tube

Telephonic interviews were carried out by one author (A MK). Physical domain of quality of life parameters was assessed with the help of Karnofsky Performance Scale [13,14]. This has five grades 0–4, 0 being fully active, able to carry on all pre-disease performance with out restriction. Grade 1 being restricted in physical strenuous activity, ambulatory and able to carry out work of a light or sedentary nature. Grade 2 is ambulatory and capable of self care, but unable to carry out any work activities, up and about more than 50% of waking hours. Grade 3 is limited self care and, confined to bed to chair more than 50% of waking hours, and Grade 4 being no self care and totally confined to bed.

Psychological and social domains of health related quality of life parameters were assessed from patients or caregivers regarding the decision of repeating or reinserting PEG-tube if needed. The advantages of the PEG tube such as; ease in feeding, cosmetic acceptability, increase in the survival period and its impact on the physical well being of the patients were also enquired. Disadvantages such as; increased time consumptions in feeding, dependency on others for feeding and any cost issues of the care were also assessed during the interview.

### Statistical analysis

The data was recorded and analyzed on SPSS version 10.0 (Chicago, IL, USA). Basic descriptive statistics including; means with standard deviation, and ranges where applicable are reported for the quantitative variables and numbers (percentages) for the qualitative variables. Chi -square test was applied to compare the proportion of difference in quality of life scores and telephonic response of the patients/care givers at p < 0.05 level of significance.

### Ethical approval

Ethical approval was obtained from the Aga Khan University Karachi, Ethical Review Committee.

## Results

### PEG-tube related outcomes

During the study period, 191 patients underwent PEG-tube placement, of those 120 (63%) were males. Over all mean age was 63 years (range 7 – 99 years). The underlying diagnosis was cerebrovascular accidents leading to feeding difficulty in 121 patients (63 %), dementia associated dysphagia in 26(14%), and others are described in Table 1.

Early complications encountered within 72 hours were; PEG-tube site infection evident by fever, collection of pus and mild excoriation in 6(3%) patients, other early complications such as bleeding and perforation were not found. Late complications (observed 72 hours after PEG-tube placement) were infection at the PEG-tube site in 29(15 %) patients, PEG-tube dislodgment/displacement in 22 (11.5%), and PEG-tube blockage in 4(2.1%) patients.

Mean follow up was found to be 365 ± 149 days; this was noted from patient's visits either in the clinic, emergency room visits, endoscopy suit or admission to the hospital. During this follow up PEG-tube remained patent for feeding. It was replaced in 43/191 patients (22.5%); single time replacement of PEG-tube was done in 26(14%) patients, and twice in nine (5 %) patients. While three and four times PEG-tube was replaced in four (2%) patients each. PEG-tube was removed altogether in 9 (5 %) patients when they were able to start per oral intake.

### Health Related Quality of Life (HRQoL) in patients with PEG-tube

Assessment of HRQoL of these PEG-tube placed patients, was possible in 126(65%) patients/caretakers; as 65 (35%) were not interviewed because they were unable to be contacted or declined to respond. Only a minority 12/126(10%) patients were able to give interview by themselves, since majority of patients were in Karnofsky score greater then 2(unable to communicate). Physical domain of HRQoL was assessed with the help of Karnofsky Performance Score. The Score of 0, 1, 2, 3 and 4 was found in 13(10%), 18(14%), 21(17%), 29(23%) and 45(36%) of patients respectively. Chi square of k-proportions was applied which showed that Karnofsky score 4 being statistically significant with p-value < 0.001 in comparison to all other grades. This signifies that majority of our patients 45(36%) were totally confined to bed and were unable to carry on self-care.

Assessment of social and psychological domains of HRQoL showed that, majority of patient/care givers; 76(60%) would like to have PEG tube again if required. Ease in feeding was noted by 105(84%) care givers/patients while dependency on others for feeding was noted by 45(36%) respondents. Moreover 62 (49%) were in the impression that feeding through PEG-tube increased the overall cost of care in such patients. Responses to other questions are given in Table 2.

## Discussion

In this study, we not only have assessed the safety and complications associated with the PEG tube, but also taken in the perspective of care givers and the patients about the PEG-tube placement. Studies related to the patients/care givers perspective regarding PEG-tube are limited. According to our literature search, this is first study which addressed the health related quality of life in patients with PEG-tube placement in the underdeveloped world It is known that PEG tube placement has a high success rate and a relatively low morbidity and procedure related mortality [15,16]. Our results also demonstrated the similar level of safety with PEG-tube placement such as infection at the PEG-tube site. Likewise, no major long- term complications were noticed such as early mortality and fistula formation in the present series of patients [17,18].

Prolongation of a poor quality of life by artificial means of nutrition is an ethical dilemma [19]. Should a poor quality of life be prolonged, the answer is not yet clear. It has been observed in a study that decision about artificial feeding in advanced dementia is a difficult task [20]. This also imposes a major financial, ethical and moral issue. Moreover in patients with dementia, after placement of PEG-tube, could be deprived of the sensation of taste, touch, nurturing and socialization [21,22]. In this context, it is worth mentioning that in the present series, majority of our responders to telephonic interview affirmed positively about prolonged survival via nutrition through the PEG-tube. They were able to handle PEG-tube quite easily and found PEG-tube more convenient then nasogastric feeding tube. Care givers 62(49%) did, express concerns regarding the financial burden, which could have attributed to replacement procedures and use of commercially available feeds.

Actual nutritional gain has now been an important issue and has gained significant attention internationally. As a result, the role of PEG-tube been debated in terms of objective nutritional benefit [22,23]. Unfortunately periodic measurements of weights of the patients, and serum albumin were not available in our series because of a retrospective nature of the study and hence that remains the main limitation of our study.

Decision of the PEG-tube insertion is a joint responsibility of the clinician, health care provider, family and if possible the patient as well [24,25]. Review of literature does support that careful judgement or a 30 day gap from the request generation for the PEG tube should be taken into consideration before making the final decision [11,26]. It was felt that, thorough explanation of the procedure and post insertion care needs to be explained at length with the care givers [24,27]. Furthermore the overall and potential gain through this procedure needs careful monitoring and types of feeds used through this tube should be assessed on a regular interval [28]. Management decisions should arise from clear determination of overall goals of care which should incorporate cultural, personal, family, spiritual and religious beliefs [28,30].

In a developing country like Pakistan, scarce resources impose an important question about the deployment of this new palliative modality for neurogeneic dysphagic patients. The per capita Gross National Income (GNI) of about $690 in 2005, as per World Bank report 2006, the World Bank considers Pakistan a low-income country [29]. For a person, placement of PEG tube being replaced a number of times poses a significant financial impact. Moreover the usage of commercial formula feeds have a financial constrains too, that are being imported from outside country, (not being manufactured in Pakistan), and many people prefer to use it as compared to home based formulas. Regarding the care takers time, he/she is involved in caring of the patient self care along with PEG tube, preparation of feed, and administering feed every 4 – 6 hours with round the clock involvement of that personnel, which is usually done by either spouse or a family member, because of hiring a nursing personnel itself would be a financial burden. These are main concerns which were brought by the care takers during the telephonic interview. The above described facts highlights the difficulties in employing PEG tube placement and therefore limits its usefulness in lower income countries such as Pakistan. It is the responsibilites of the Physician to provide a realistic picture to family and care givers about expected potential benefits, the morbidity associated with the PEG-tube placement, its impact on the quality of life and overall health status of these patients [30].

## Conclusion

The present series reaffirms that PEG-tube placement is relatively free from serious immediate and long- term complications and an acceptable modality. Majority of caregivers and patient felt PEG-tube helped in prolonging the survival and helpful in feeding. The real benefit in terms of quality of life is still doubtful and an ethical dilemma. Studies are needed to assess the actual nutritional gain objectively in these patients especially in setting of lower income countries.

## Competing interests

The author(s) declare that they have no competing interests.

## Authors' contributions

SA conceived the idea, M KA and SA designed the study and questionnaire, MKA conducted the study questionnaire and reviewed the charts, WJ, ZA, HS, SH, RW participated in study design and coordinated the study. All authors read and approved the final manuscript.

## Pre-publication history

The pre-publication history for this paper can be accessed here:



## Figures and Tables

**Table 1:** Underlying Diseases in patients who had PEG-tube placed (n = 191)

Aetiology	n (%)
Dysphagic Stroke	121(63)
Dementia	26(14)
Advanced Parkinsonism	21(11)
Oropharyngeal tumours	14(7)
CNS tumours	5(3)
Olivopontine cerebellar atrophy	2(1)
Wilson's Disease	2(1)

Results are presented as numbers (percentage)

**Table 2:** Care giver/patients perspective regarding PEG-tube placement. (n = 126)

Question	Yes n (%)	No/indecisive n (%)	p-value
Would you like to have PEG tube again if required?	76 (60)	50 (40)	0.021
Ease in feeding	105 (84)	21 (16)	< 0.001
Cosmetically acceptable	79 (63)	47 (37)	0.004
PEG tube increases the survival	76 (60)	50 (40)	0.021
Increase time consumption due to feeding	49 (39)	77 (61)	0.013
Dependent on others for feeding	45 (36)	81 (64)	0.001
Increase in the cost of care	62 (49)	64 (51)	0.859

Results are presented as numbers (percentages) and p-value < 0.05 was considered statistically significant by applying chi-square test of k-proportion.
